# Mass Density Characterization of Hydrogel-Based Systems Inoculated with Bacterial Strains and Dose-Response Performance of *Escherichia coli* Inoculation

**DOI:** 10.3390/jfb16040121

**Published:** 2025-04-01

**Authors:** Carolina Salinas Domján, Mauro Valente, Marcelo R. Romero

**Affiliations:** 1 Instituto de Física Enrique Gaviola (IFEG), CONICET-UNC, Córdoba X5000HUA, Argentina; 2 Laboratorio de Investigación e Instrumentación en Física Aplicada a la Medicina e Imágenes de Rayos X (LIIFAMIRx), FaMAF-UNC, Córdoba X5000HUA, Argentina; 3 Centro de Excelencia en Física e Ingeniería en Salud & Departamento de Ciencias Físicas, Universidad de la Frontera, Temuco 4811230, Chile; 4 Facultad de Ciencias Químicas, UNC, Córdoba X5000HUA, Argentina; 5 Instituto de Investigación y Desarrollo en Ingeniería de Procesos y Química Aplicada, IPQA-CONICET, Córdoba X5016GCA, Argentina

**Keywords:** hydrogel-based materials, *Staphylococcus aureus*, *Escherichia coli*, cell culture media mass density, spectrophotometry, ionizing radiation dosimetry

## Abstract

This study aims to determine the density of two hydrogel-based media, medium with agar-agar and medium with agar-agar and glucose, which are suitable for both irradiation and bacterial growth, considering the presence or absence of *Staphylococcus aureus* and *Escherichia coli* strains. The viability of Escherichia coli cell-inoculated systems was also evaluated to explore potential applications in radiation dosimetry within the 0–10 Gy range, using spectrophotometric and bacterial culture methods. Mass density measurements were performed at varying temperatures using two approaches: the first one, based on direct measurements of mass and volume, yielded densities comparable to liquid water, with uncertainties ranging from 9 to 16%, while the second approach, employing Archimedes’ principle (mass in air vs. mass in a liquid of known density), produced more accurate results, with uncertainties between 0.04 and 0.08%, thus proving more reliable for density determinations. Furthermore, the feasibility study of Escherichia coli-inoculated systems for ionizing radiation dosimetry demonstrated a linear spectrophotometric response to radiation doses across the investigated range, particularly for samples stored at 25 °C. The studied systems were characterized in terms of the corresponding growth curve and post-irradiation bacterial survival, supporting their potentiality as reliable ionizing radiation dosimeters.

## 1. Introduction

The constant development of highly sophisticated techniques in both diagnostic and therapeutic procedures using ionizing radiation has shown a growing demand for reliable and accurate dosimetry techniques. Innovative high performance dosimetry methods require materials capable of determining absorbed dose, even when information on energy and nature of charged particles at the point of interest is incomplete or fragmentary [1].

On the one hand, novel radiosensitive materials may be used to simulate human tissues and organs, commonly referred to as tissue-equivalent materials [2,3,4,5,6,7,8], or to determine absorbed dose in water following the international dosimetry protocols TRS-398 IAEA and TG-51 AAPM, referred to as water-equivalent materials [9,10,11,12,13]. Selecting an appropriate dosimetry material requires comprehensive information about its properties, ideally matching the absorption and dispersion characteristics of the target material to be emulated. Gel dosimeters emerge as the most suitable option for designing and implementing dosimeters capable of performing direct measurements of absorbed dose in aqueous media [10].

International ionizing radiation dosimetry protocols recommend both instrumental and measurements’ calibration be expressed in terms of absorbed dose in liquid water, under normal temperature/pressure conditions [1,14]. Therefore, it is desirable to develop dosimeters with sensitive volumes made of materials with radiation interaction properties close to those of liquid water, so that correction factors may not be necessary and beam perturbation is avoided during the irradiation. Furthermore, water-equivalence dosimeters are necessary to enable consistent benchmarking against reference dosimetry protocols and to enhance the reproducibility of dose measurements across varying radiation fields and energies. In this regard, if two materials have similar mass absorption/scattering properties for photon and charged particles’ beams over a certain energy range, then the corresponding particle transport and further dose deposition for both materials will be similar [15]. Hydrogels are three-dimensional (3D) polymer networks that are strongly imbibed by water, whose amount approaches up to 99% *w*/*w* of the total hydrogel mass [16]. Thus, in terms of the radiation–matter interaction, compounds that are candidates for dosimetry materials can be considered water-equivalent if the corresponding fundamental physical quantities, such as the mass attenuation coefficient ($\mu /\rho =ln(I_{0}/I)/\rho x$) and the mass stopping power (S=−dE/ρdx), are consistent (typically, less than 1% deviation is accepted) with those of liquid water–considered as the reference material–within the energy range of interest [1,2,10]; $I_{0}$ stands for the incident intensity that penetrates a material of mass density ρ and thickness *x* and *I* is the emerging intensity; while *E* is the average energy dissipated per unit of traveled length in the compound.

Hence, proper assessment of the bulk mass density (ρ) of materials of dosimetry interest is extremely important as it allows for establishing the radiation–matter interaction properties required for radiological applications, as previously proposed by many authors through multiple approaches [17,18,19,20,21,22].

However, traditional methods applied to dosimetry primarily involve passive or inert systems with physical or chemical detectors, which are highly effective for quantifying exposure but fall short in providing real-time biological information and understanding the nuanced effects of ionizing radiation at the cellular and molecular levels. Within the radiobiology framework, understanding how ionizing radiation affects living systems (or tissues) is essential for developing better determination of the early and late biological effects of ionizing radiation.

Additionally, in the search for innovative approaches to developing systems with high tissue equivalence, the use of microbiological agents distributed in hydrogels is proposed. Bacteria have shown promise as tools in whole-cell biosensors, leveraging their biochemical complexity [23] for applications such as bioremediation tasks [24] and even as therapeutic agents in the fight against cancer [25].

In this context, combining the metabolic activity of microbiological agents with gel-based dosimetry characteristics [10,26,27,28] emerges as an innovative approach, using biotechnology and molecular biology procedures as an unconventional alternative to develop a whole-cell sensor. This approach also represents a potential method to achieve 3D dose mapping within a biological environment. Consequently, the proposed sensor utilizes bacterial culture media capable of characterizing the absorbed dose through the survival of these microorganisms [29].

Therefore, this study aims to determine the mass density of two hydrogel-based cell culture media: (*i*) Medium with Agar-Agar (MAA) and (*ii*) Medium with Agar-Agar and Glucose (MAAG) [29], which are suitable for both irradiation and bacterial growth, considering the presence or absence of *Staphylococcus aureus* (SA) and *Escherichia coli* (EC) strains. Thus, accurate assessments of the bulk mass density of the proposed hydrogel-based bacteria-infused dosimeters have been performed as key information that leads to similarities/discrepancies in radiation attenuation/scattering properties and thereby ultimately affecting the accuracy of dose measurements. Additionally, considering that SA-inoculated systems have already been investigated in relation to the corresponding dose-response performance [29], the viability of EC-inoculated systems is evaluated for its potential applications in radiation dosimetry within the 0–10 Gy range, using simple analytic techniques such as spectrophotometric and bacterial culture methods.

This integration of microbiological agents with gel-based dosimetry characteristics may offer the potential to capture biological responses to ionizing radiation within hydrogel systems capable of conforming to specific structures (such as the shape and size of soft tissue organs), providing insights into immediate and long-term effects, and furnish critical data directly applicable to radiobiological models, which traditional passive or inert dosimetry methods are unable to provide. The proposed systems have the potential to significantly contribute to radiation dosimetry by enabling the direct measurement of biological responses to radiation exposure.

## 2. Materials and Methods

### 2.1. Culture Media and Bacterial Strains

Culture media are intended as a set of essential nutrients that create the conditions for the development of the microorganisms under study. Based on previous publications [29,30], agar-agar has been used as the main component and support matrix for culture media solutions. Sodium chloride (NaCl) is also added to the solution to promote an isotonic concentration, fostering conditions similar to those of biological tissues and optimal for microbial growth. In order to provide sufficient nutrients to promote proper bacterial growth, agar PCA is used, forming the first medium (MAA), while the second developed medium, called MAAG, contains a glucose addition to the MAA formulation. Both compositions are summarized in Table 1 [29].

The media are studied with and without inoculated bacterial content within them to develop systems with high tissue equivalence by using bacteria distributed in hydrogels, since the proportionality between the absorbed dose due to X-rays and the number of colony-forming units has been demonstrated [31].

**Figure 1:**
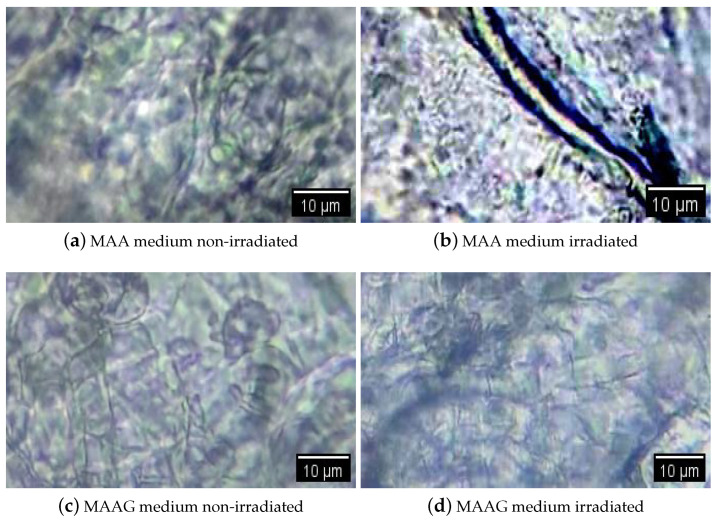
Microscopic images (1000×) of the studied media without inoculation. MAA medium, non-irradiated (**a**), irradiated at 30 Gy (**b**), MAAG medium, non-irradiated (**c**) and irradiated at 30 Gy (**d**), respectively. The samples were lyophilized prior to their visualization.

Figure 1 shows an example of visualization through an optical microscope (WJTQTBT, binocular microscope W-120) of the proposed systems without inoculation, with two samples irradiated at 30 Gy and two non-irradiated samples. Prior to their microscopic visualization, all samples were lyophilized [32]. Thus, the implemented process consisted of taking a fragment of approximately 5 mm diameter per sample and placing it in an Eppendorf tube. Liquid air was then placed in a beaker and the samples were immersed for 5 min with the lid open. Subsequently, a cover was formed over each Eppendorf tube and the surface of the aluminum paper was pierced with a hypodermic needle. The tubes with the frozen samples were placed in the pre-cooled container of the lyophilizer (L-T8, RIFICOR, Argentina) at −44 °C and vacuum was created to reach 0.022 mmHg. The samples were lyophilized under these conditions for 8 h and, after completion of the procedure, the aluminum caps were removed and the tubes were sealed for analysis under the optical microscope. It can be observed that the captured microscope images show few structural changes in the samples upon irradiation. Among them, the MAAG matrices were the least modified by X-rays; this may be due to the presence of glucose in the medium, which serves to capture free radicals. This is known in the literature, as shown in [33]. On the other hand, some slight alterations have been observed in MAA samples; however, they require more in-depth studies in microscopy as well as the analysis of changes in the network and diffusion parameters to confirm such assertions.

The first bacterial strain used is *Staphylococcus aureus* (SA) ATCC^®^ 25923, an anaerobic, Gram-positive, coagulase- and catalase-positive, motionless and non-sporulating bacterium. Its diameter ranges from 0.5 to 1.5 μm and tends to form grape-like clusters [34].

The second strain used is *Escherichia coli* (EC) ATCC^®^ 25922. EC is a Gram-negative bacillus approximately 1 μm long and 0.35 μm wide, although it may vary depending on the strain and its conditions. It may have flagella operating as whip-like structures for mobility or pili for surface adherence. Physiologically, it is an facultative anaerobe capable of growth with or without oxygen, but cannot grow at extreme thermal or pH conditions [35]. Phylogenetically, it belongs to the Enterobacteriaceae family and is closely related to pathogens such as Salmonella, Klebsiella, and Serratia [36].

### 2.2. Culture Media’ Preparation and Inoculation

Based on the concentrations shown in Table 1, the media elaboration process consists of suspending the powdered solids in sterile distilled water, leaving them to stand for 5 min, and then heating them with constant stirring until boiling for complete dissolution. Once finished, the medium is sterilized in an autoclave with water at 121 °C for 15 min. For those samples designated for inoculation, their respective medium is prepared with a reduction of 1 mL in water.

Once the sterilization has finished, those volumes that are not to be inoculated are poured into vials of 1 × 1 × 2 cm^3^, cataloged, sealed with parafilm, and stored at 4 °C for at least 8 h with the volumes designated to be inoculated. Subsequently, the media to be inoculated are brought to a water bath between 50 to 55 °C to prevent gelation before inoculation.

**Figure 2:**
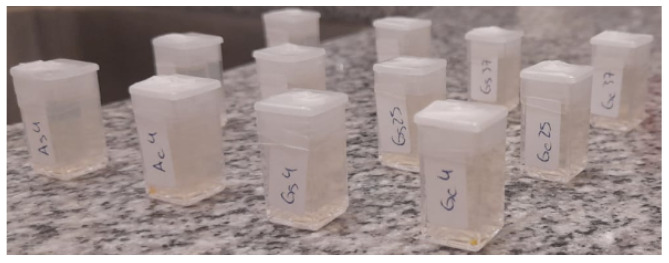
Elaborated samples to be further studied.

For each bacterial strain, a vial is prepared containing 1 mL of sterilized distilled water at the same temperature of the water bath and inoculated with one loopful of the respective strain. Once homogenized, the volumes of MAA and MAAG are inoculated accordingly, poured into cataloged vials and sealed with parafilm. A total of 18 samples were prepared, as reported in Figure 2.

The density test is performed for each medium, with and without bacteria, at 5, 25 and 37 °C. Once the samples are prepared, each is maintained at its designated temperature for at least 24 h. For streamlined measurement processing, each sample is cataloged as indicated in Table 2.

### 2.3. Mass Density Measurements

Density measurements for each medium in the presence and absence of bacteria at different temperatures are carried out using two different approaches. It should be noted that since the water content in each medium is higher than 96% w/w, densities are expected to approximate that of liquid water.

The first approach consists of direct mass and volume measurements of each sample. Mass measurements are performed with Mettler Toledo ME204 (Mettler-Toledo S.A.E., España) analytical balance, while volume measurements are carried out by recording the lengths of each side of the cube that conforms the sample with a ±0.1 cm precision rule. Each measurement is made at least three times per sample, from which the average is calculated and the density is determined by using the following equation:

(1) $$\rho =\frac{m}{V},$$

where ρ is the density, and *m* and *V* are the mass and volume of the sample, respectively.

**Figure 3:**
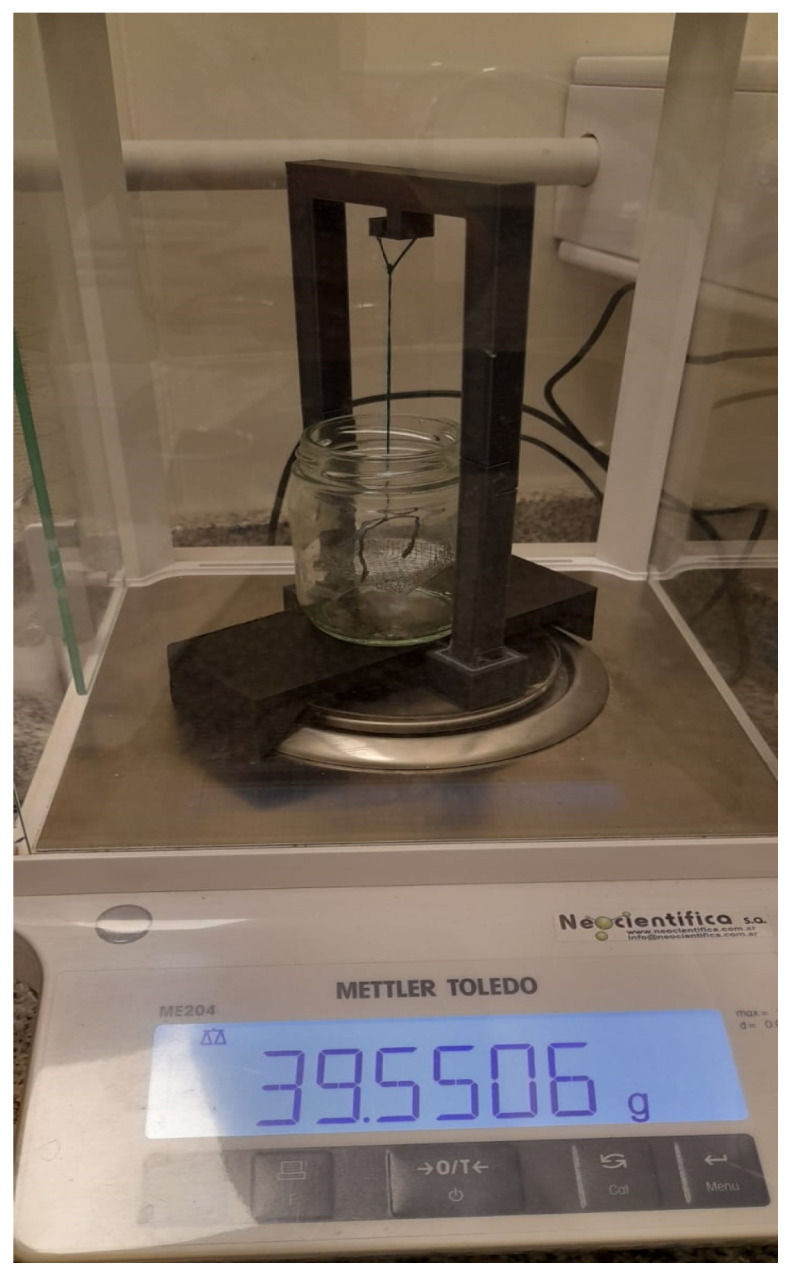
Setup to assess mass density by means of the Archimedes’ principle.

For the second approach, using the setup shown in Figure 3, the density of the media is determined based on the Archimedes’ principle.

The procedure is as follows: Firstly, it has to be ensured that the balance is calibrated and zeroed. Then, the mass in air ($m_{1_{c,a}}$) of the basket that will hold the sample and the mass in air of the basket containing the sample are weighed ($m_{1_{mc,a}}$). Subsequently, the mass of the basket submerged in a liquid with known density is performed ($m_{2_{c,h}}$)—in this case, heptane with a density $\rho_{heptane}=0.710$ g/cm^3^ has been used. Finally, the mass of the basket containing the sample immersed in heptane ($m_{2_{mc,h}}$) is established.

Once these data are known, it is possible to calculate the mass of each sample in air ($m_{m_{a}}$) and immersed in heptane ($m_{m_{h}}$), as well as the thrust mass ($m_{e}$), from which it is possible to know the volume of the sample ($V_{m}$), and finally, determine its density ($\rho_{m}$). Equations (2)–(6) detail the calculations for each of these parameters. A minimum of three measurements are performed per sample to ensure accuracy.

(2) $$m_{m_{a}}=m_{1_{mc,a}}-m_{1_{c,a}}$$

(3) $$m_{m_{h}}=m_{1_{mc,h}}-m_{1_{c,h}}$$

(4) $$m_{e}=m_{m_{a}}-m_{m_{h}}$$

(5) $$V_{m}=\frac{m_{e}}{\rho_{heptano}}=\frac{m_{m_{a}}-m_{m_{h}}}{\rho_{heptano}}$$

(6) $$\rho_{m}=\frac{m_{m_{a}}}{V_{m}}=\frac{m_{m_{a}}}{m_{m_{a}}-m_{m_{h}}}\cdot \rho_{heptano}$$

It is worth remarking that maintaining the heptane at the same temperature as the samples during density measurements at different temperatures is mandatory.

### 2.4. Microbial Growth Feasibility and Characterization

After determining the density of the corresponding media, to assess whether the strain can grow effectively in each proposed medium, two Petri dishes are prepared with MAA or MAAG medium, respectively, and the EC strain is cultured. The plates are incubated for 24 h at 37 °C.

If growth is confirmed, its characterization follows a procedure similar to that in [29]. Each culture media batch is separated into two volumes, V1 and V2, where V1 corresponds to the non-inoculated samples and is poured into blank vials, while V2 corresponds to the culture material to be inoculated. The prepared samples are initially stored at 4 °C for at least 8 h. Subsequently, the blank samples are brought to their respective temperatures, while V2 is heated in a water bath at 55 °C with constant stirring and inoculated by adding 1 mL of distilled and sterilized water, at the same temperature, containing 2 mg of EC microbiological content per 15 mL of media. Once homogenized, the preparation is poured into vials to be stored at 4, 25, and 37 °C, respectively.

A total of 3 blank vials and 12 inoculated vials are prepared per media. Absorbance measurements are performed with a UNICO S1205 spectrophotometer at 800 nm every 15 min after inoculation.

### 2.5. Dose-Response Preliminary Performance

**Figure 4:**
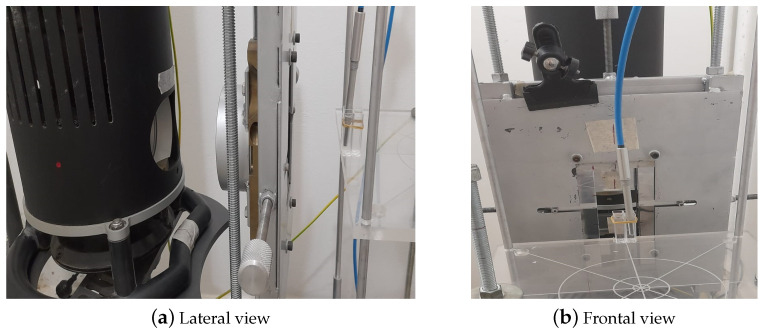
Example of sample position and calibration with ionization chamber.

The absorbed dose and the absorbed dose rate values were correctly established using a calibrated ionization chamber system consisting of a Farmer PTW-type ionization chamber, a UNIDOS electrometer, and the corresponding extension cable, together with the calibration certificate for soft X-ray beams up to 30 kVp. The water-proof ionization chamber was placed inside a spectrophotometry vial filled with distilled water to adequately represent the irradiation conditions of the test samples, as shown in Figure 4.

With the exception of specialized radiotherapy modalities, such as stereotactic radiosurgery, conventional radiotherapy treatments typically involve absorbed doses per session of up to 10 Gy [37]. As a result, the dose-response characterization of dosimetry systems designed for radiotherapy applications is commonly performed within this dose range. Therefore, a preliminary evaluation of the dose-response performance of the proposed bacteria-inoculated dosimeters was conducted by irradiating samples through gelled media with microbiological content in the stationary phase under the following conditions: 3 samples serve as blanks (non-irradiated), 3 samples are irradiated at low doses (1.00 ± 0.02 Gy), 3 samples are irradiated at an intermediate dose level (5.0 ± 0.2 Gy), and 3 samples are irradiated at a high dose (10.0 ± 0.2 Gy). Once irradiated, the samples from each group are stored at different temperatures: 4, 25, and 37 °C.

The samples were irradiated using an orthovoltage X-ray tube available at the LIIFAMIR^x^ laboratory facilities. A conventional 1 kW power tube (YXLON EVO 225) [38] equipped with a tungsten anode has been configured to operate at 150 kV, 6.0 mA to irradiate samples placed at a source–surface distance (SSD) of 36.0 ± 0.5 cm. Absorbance measurements are recorded before irradiation of the sample, and post-irradiation measurements are carried out during the first 3 h as well as 24 h and 48 h after irradiation in order to control possible variations over time.

## 3. Results and Discussion

This section is organized according to the proposed scopes reporting the mass density of culture media in the first instance, followed by the characterization of microbial assay growth, and finally, a preliminary approach to the corresponding overall dose response to X-ray radiation.

As is known, complex modern radiation therapy modalities involving mixed radiation fields, like boron neutron capture therapy and hadrontherapy, present new challenges to dosimetry techniques in assessing the reliable characterization of therapeutic dose components by means of tissue-equivalent systems. In this context, and to the best of the authors’ knowledge, the dosimetry systems described in this study are currently not available. This highlights the significance and potential of the present study.

### 3.1. Culture Media Mass Density

#### 3.1.1. Mass Density Assessment According to the First Measurement Approach

The results obtained from the first approach are shown in Table 3, where the average mass and volume measured for each medium and corresponding density are reported. Uncertainties were calculated from error propagation.

Notably, no significant mass density differences have been found between media with and without bacteria according to the corresponding uncertainties. This trend might be expected given that the variations are due to a combination of the presence or absence of bacteria and the presence or absence of glucose. Furthermore, since the elemental composition of bacteria generally includes mainly carbon, hydrogen, oxygen, and nitrogen, which are predominant elements in chemical/polymer dosimetry gels, the differences concerning them are comparable.

Among the most commonly used chemical/polymer gel dosimeters for ionizing radiation dosimetry are Fricke gel [39] and polymeric gel dosimeters [28] such as gels based on N-isopropylacrylamide (NIPAM), polyacrylamide (PAGAT), and itaconic acid (ITABIS), which have been shown to be water-equivalent [9,10]. These known dosimeters have the capacity to quantify the absorbed dose and measure the spatial distribution of radiation through different mechanisms. Fricke gel dosimeters use the radiation-induced conversion of ferrous ions (Fe^2+^) into ferric ions (Fe^3+^), where the amount of ferric ions produced is directly proportional to the dose of absorbed radiation [9,39]. On the other hand, polymeric gel dosimeters induce a polymerization reaction proportional to the absorbed dose due to their chemical reagents, which are sensitive to radiolysis-produced free radicals [9,10,28]. Due to the relevance of these already known and widely used dosimetric systems, their densities are compared with the proposed MAA and MAAG systems.

**Figure 5:**
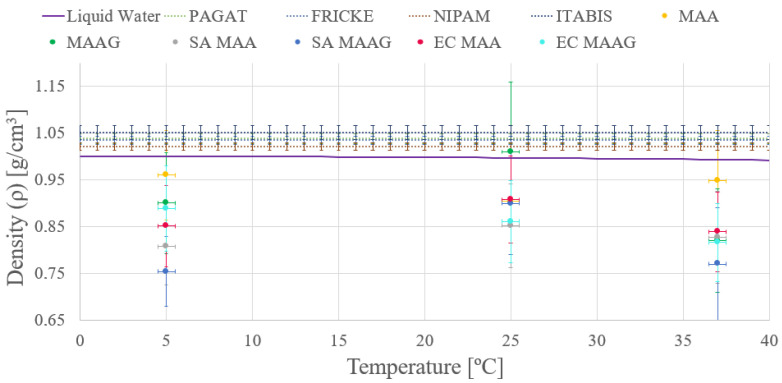
Temperature depending on the bulk mass density as compared to the liquid water and well-established polymer gel dosimeters at 25 °C (extended with dashed line) according to the first approach.

As depicted in Figure 5 both media present enhanced density in the absence of bacteria, a trend consistent across both the studied strains. However, given the uncertainties—ranging from 9 and 16% of the final value obtained—stemming from the applied measurement methodology, conclusions based on this trend may be premature, a feature that will be reassessed after reviewing the results of the second measurement approach. Additionally, the mass density of the dosimetry gels is comparable to that of liquid water, as widely reported in the literature [10,40,41].

Moreover, another remarkable observation is that the density of both media varies with temperature.

#### 3.1.2. Bulk Mass Density Assessment According to the Second Measurement Approach

The results obtained from the second approach are summarized in Table 4, presenting the values of mass in air ($m_{m_{a}}$) and mass immersed in heptane ($m_{m_{h}}$), along with the values obtained for volume ($V_{m}$), mass density ($\rho_{m}$), and corresponding uncertainties.

**Figure 6:**
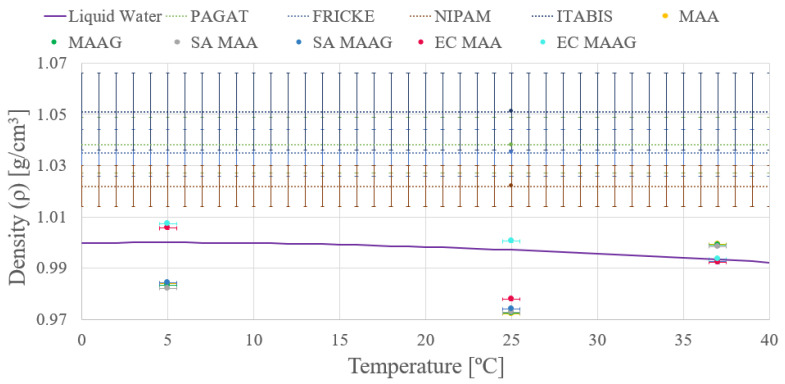
Temperature depending on the bulk mass density as compared to the liquid water and well-established polymer gel dosimeters at 25 °C (extended with dashed line) according to the second approach.

Similar to the findings of the first approach, it can be noted that the final density values do not exhibit significant differences. However, the second approach shows enhanced precision in both measurements and density calculations, as may be appreciated in Figure 6, where almost negligible variations are shown between the media with and without microbiological content. Data are reported for both liquid water and different dosimetry gels.

Conversely, it is evident that while the obtained mass density values do not coincide with the liquid water within corresponding uncertainties, they are closer than those obtained through the first approach. In addition, the uncertainties achieved from the second approach vary between 0.04 and 0.08%, which are significantly lower than the uncertainties obtained from the first approach.

Thus, the second approach and its results are considered for guiding future tests and studies, whether from an experimental, theoretical, or computational point of view.

**Figure 7:**
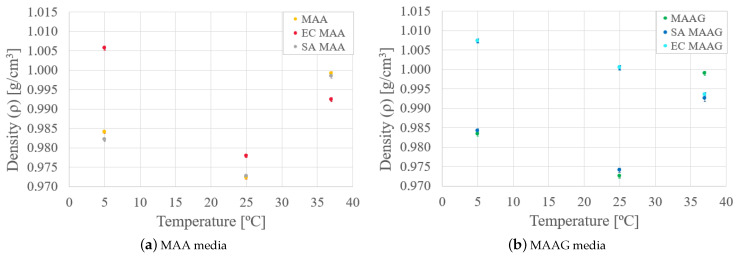
Bulk mass density variations as a function of the temperature, as obtained by the second approach, for MAA (**a**) and MAAG (**b**) media.

Finally, by analyzing the mass density behavior of each medium in the presence and absence of bacteria, as reported in Figure 7, it is possible to reaffirm that mass density varies as a function of temperature for both media. In the case of the SA strain, the behavior is similar to that of the media without microbiological content, while with the EC strain, greater variations occur, especially at lower temperatures, where the density is higher.

Likewise, as the temperature increases for both media and strains, there is generally an enhanced similarity between samples with respect to their mass density values.

It should be noted that the low density variation observed in the materials of interest, under the conditions studied, implies a lower effect on the mass attenuation coefficient and stopping power [1,2,10] if the materials are compared in terms of the radiation–matter interaction. Furthermore, by presenting densities similar to water, it could be preliminarily inferred that the proposed materials might present high tissue equivalence if low-density (soft) tissues are considered [42,43].

### 3.2. Microbial Growth Characterization

**Figure 8:**
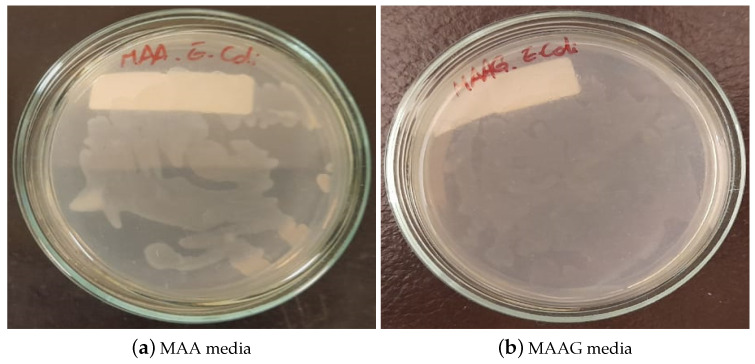
*Escherichia coli* growth on Petri dish for MAA (**a**) and MAAG (**b**) culture media, after 24 h of incubation.

After 24 h of cultivating and incubating the EC strain for the evaluation of the feasibility of the strain growth in each proposed medium, the results were visually inspected. As illustrated in Figure 8, growth is confirmed in both media, highlighting their suitability for microbiological growth, with notable differences, such as MAAG presenting greater growth compared to MAA. This result aligns with expectations, since the presence of glucose in MAAG serves as a primary energy source for microorganisms [44], thus facilitating more effective bacterial growth.

**Figure 9:**
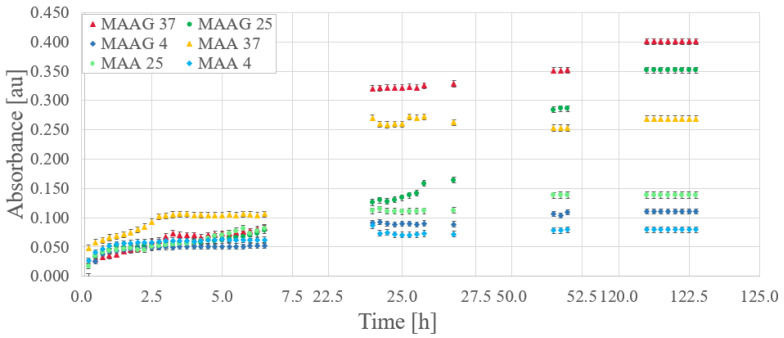
Absorbance as a function of time at different temperatures for MAA and MAAG culture media.

Based on preliminary characterizations of the proposed culture media [29], optical absorbance measurements were performed in triplicate for EC samples inoculated at 800 nm, with readings taken at 15-min intervals. Figure 9 reports the bacterial growth obtained for each culture media. Gaps between data (missing points) correspond to periods at night or during weekends, when measurements were not feasible.

Relevant considerations arise when comparing storage temperatures for the same growing medium. Direct inspections reveal that while initial bacterial growth is robust at all temperatures, over time, samples stored at higher temperatures exhibit higher absorbance, while those stored at 4 °C demonstrate lower growth compared to those stored at 25 or 37 °C. Unlike the results reported for the SA strain, where the greatest growth after a period of time occurs at lower temperatures [29].

However, the samples corresponding to the MAAG media present a higher growth rate, favored by the presence of glucose. Table 5 displays the percentage differences in growth between the two media for different temperatures with the EC strain, which is also illustrated in Figure 9. Notably, as time increases, percentage differences become more pronounced for the MAAG medium as compared to the MAA media at the same temperature. Although the highest final growth is reported at a temperature of 37 °C, the highest percentage differences in growth between the media occur at 25 °C, reaching a value of 153%, indicating that at this temperature, the presence or absence of glucose in the media has a significant impact.

It should be noted that the percentage differences in growth between media exceed the 25% reported for the SA strain in all cases.

### 3.3. Preliminary Dose-Response Performance of Hydrogel-Based Dosimeters Infused with Escherichia coli

**Figure 10:**
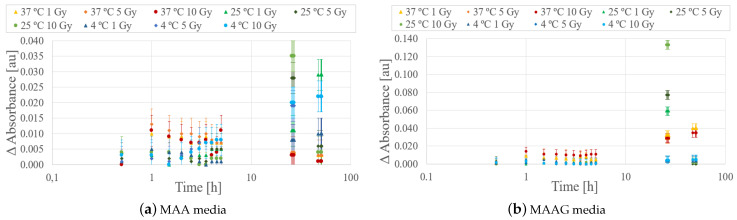
Absorbance differences (ΔAbs.) as a function of time for each culture medium, MAA (**a**) and MAAG (**b**), at different temperatures.

Absorbance measurements were carried out on each sample before irradiation, immediately after, and over a post-irradiation period of 50 h. The response of samples stored at 4, 25, and 37 °C was evaluated by exposing samples to doses of 1, 5, and 10 Gy for each medium. As illustrated in Figure 10 on a logarithmic scale, the dose–response relationship of the EC strain during the initial 10 h post-irradiation exhibits no discernible correlation between the system’s response and the absorbed dose, suggesting a non-significant association between these two variables. However, noticeable changes appear after 24 h, contrasting with the observations made for the SA strain [29], which displayed an evident correlation in the dose response within 2 h after irradiation at a specific temperature.

During the measurement process, the base vial is defined as the vial containing non-irradiated bacteria, and the changes in recorded absorbance take into account not only the medium’s composition and variations but also the growth and survival of the bacteria.

**Figure 11:**
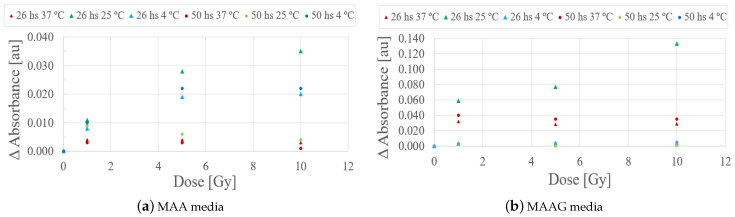
Absorbance differences (ΔAbs.) as a function of dose for each medium, MAA (**a**) and MAAG (**b**), at different temperatures.

Evaluating the behavior of the studied samples at 26 and 50 h after irradiation, as illustrated in Figure 11, it can be quoted that the samples stored at 4 and 37 °C did not exhibit significant responses that could eventually be associated with a potential low sensitivity for the studied dose range. However, samples stored at 25 °C preliminarily showed a notable response to irradiation within a period of 26 h. However, this trend could not be detected after 48 h.

**Figure 12:**
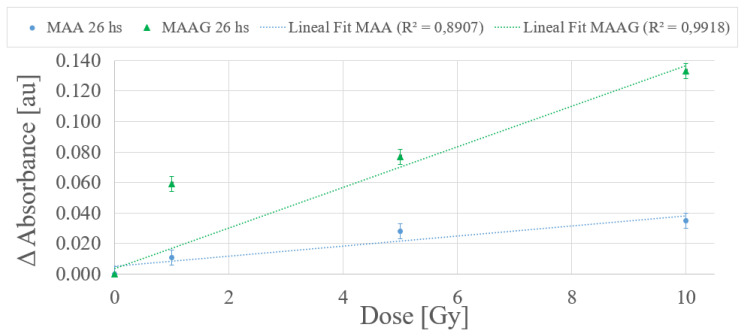
Dose-response curves within the dose range from 0 to 10 Gy.

According to the dose-response trend obtained at 25 °C, a first-order (linear) function has been proposed to fit the data for the dose range from 0 to 10 Gy for each medium. Figure 12 depicts a typical example within the dose range of interest, with measurements carried out 26 h after irradiation. The values obtained from the linear fit correlation parameter ($R^{2}$) support the proposed first-order approximation for the dose-response dependence, which aligns with the commonly desirable characteristic for any dosimetry system. Thereby, it is considered that measurements obtained between 24 and 27 h after sample irradiation demonstrate an univoque and evident response to irradiation, unlike the 2 and a half to 3 and a half hours previously reported for the SA strain.

The results reported in Figure 12, although preliminary, may assist in verifying whether there is indeed an unequivocal correlation response to the dose. Further studies for each system incorporating shorter dose intervals within the same range may be performed.

## 4. Conclusions

The present work assessed the bulk mass density of two hydrogel-based cell culture media that can be used to elaborate bacteria-infused dosimeters—(*i*) Medium with Agar-Agar (MAA) and (*ii*) Medium with Agar-Agar and Glucose (MAAG)—demonstrating also that both gels are suitable for radiation dosimetry and bacterial growth, considering the presence or absence of *Staphylococcus aureus* (SA) and *Escherichia coli* (EC) strains. Bulk mass density assessments were successfully conducted at different temperatures using two independent approaches, the first of which, based on direct measurements of mass and volume, yielded densities comparable to those of liquid water, with uncertainties ranging between 9 and 16%, while the second approach, employing Archimedes’ principle, produced more precise estimations, with uncertainties between 0.04 and 0.08%, thus highlighting the second approach as more reliable for bulk mass density determinations of bacteria-infused hydrogels.

The minor bulk mass density differences with respect to liquid water observed in the investigated materials indicate promising performance in terms of almost negligible effects on the correction requirements of the mass attenuation coefficient and stopping power for ionizing radiation dosimetry purposes. Furthermore, with a bulk mass density similar to that of liquid water, it could be preliminarily concluded that the proposed bacteria-infused hydrogel-based materials attain high water (soft tissue) equivalence. In addition, the viability of the systems inoculated with *Escherichia coli* was evaluated for their potential application in X-ray radiation dosimetry within the range of 0 to 10 Gy. The preliminary results for the dose-response output demonstrated for the Escherichia Coli-infused culture media a noticeable high linear correlation with X-ray radiation doses over the entire studied range, particularly for samples stored at 25 °C. In summary, the studied systems based on hydrogels infused with *Escherichia coli* (EC) strains were properly characterized in terms of the corresponding growth curve and post-irradiation bacterial survival, supporting their potential as effective dosimeters. Ongoing and future work will allow for expanding the present results.

## Figures and Tables

**Table 1:** Concentrations of each studied media [29].

Component	Supplier and Characteristics	Concentration	
		MAA	MAAG
Water	Distilled and sterilized	96.947% *w*/*w*	96.446% *w*/*w*
Agar Agar	Pura química	2.057% *w*/*w*	2.047% *w*/*w*
NaCl	Sigma Aldrich	0.135 M	0.134 M
Glucose	Cicarelli	-	0.484% *w*/*w*
PCA Agar	Britania Lab	0.229% *w*/*w*	0.228% *w*/*w*

**Table 2:** Sample catalog for density study.

MAA Media			MAAG Media		
Strain	Temperature [°C]	Catalog	Strain	Temperature [°C]	Catalog
Without bacteria	5	MAA 5	Without bacteria	5	MAAG 5
	25	MAA 25		25	MAAG 25
	37	MAA 37		37	MAAG 37
*Staphylococcus aureus*	5	SAMAA 5	*Staphylococcus aureus*	5	SAMAAG 5
	25	SAMAA 25		25	SAMAAG 25
	37	SAMAA 37		37	SAMAAG 37
*Escherichia coli*	5	ECMAA 5	*Escherichia coli*	5	ECMAAG 5
	25	ECMAA 25		25	ECMAAG 25
	37	ECMAA 37		37	ECMAAG 37

**Table 3:** First approach’ results for sample mass, volume, and corresponding density. As shown in Table 2, MAA and MAAG refer to medium with agar-agar and medium with agar-agar and glucose, respectively. Samples with SA indicate that the corresponding medium was inoculated with the *Staphylococcus aureus* strain, while EC corresponds to media inoculated with *Escherichia coli*. Each sample was stored at 5, 25, or 37 °C, as indicated by the number next to its specification.

Sample	Mass [g]	Volume [cm^3^]	ρ [g/cm^3^]
MAA 5	0.5759 ± 0.0004	0.60 ± 0.06	0.960 ± 0.096
MAA 25	0.5432 ± 0.0004	0.60 ± 0.06	0.905 ± 0.091
MAA 37	0.5027 ± 0.0004	0.53 ± 0.06	0.948 ± 0.107
SAMAA 5	0.4843 ± 0.0004	0.60 ± 0.06	0.807 ± 0.081
SAMAA 25	0.4854 ± 0.0004	0.57 ± 0.06	0.852 ± 0.090
SAMAA 37	0.4136 ± 0.0004	0.50 ± 0.06	0.827 ± 0.099
ECMAA 5	0.5918 ± 0.0004	0.70 ± 0.07	0.851 ± 0.087
ECMAA 25	0.6356 ± 0.0004	0.70 ± 0.07	0.908 ± 0.093
ECMAA 37	0.5660 ± 0.0004	0.68 ± 0.07	0.839 ± 0.085
MAAG 5	0.4504 ± 0.0004	0.50 ± 0.06	0.901 ± 0.108
MAAG 25	0.4036 ± 0.0004	0.40 ± 0.06	1.01 ± 0.15
MAAG 37	0.4507 ± 0.0004	0.55 ± 0.07	0.82 ± 0.11
SAMAAG 5	0.4522 ± 0.0004	0.60 ± 0.06	0.754 ± 0.075
SAMAAG 25	0.4480 ± 0.0004	0.50 ± 0.06	0.90 ± 0.11
SAMAAG 37	0.3087 ± 0.0004	0.40 ± 0.06	0.77 ± 0.12
ECMAAG 5	0.5823 ± 0.0004	0.66 ± 0.07	0.889 ± 0.091
ECMAAG 25	0.5297 ± 0.0004	0.62 ± 0.06	0.861 ± 0.088
ECMAAG 37	0.5714 ± 0.0004	0.70 ± 0.07	0.816 ± 0.083

**Table 4:** Second approach’ results for sample mass, volume, and corresponding bulk mass density. As shown in Table 2, MAA and MAAG refer to medium with agar-agar and medium with agar-agar and glucose, respectively. Samples with SA indicate that the corresponding medium was inoculated with the *Staphylococcus aureus* strain, while EC corresponds to media inoculated with *Escherichia coli*. Each sample was stored at 5, 25, or 37 °C, as indicated by the number next to its specification.

Sample	$m_{m_{a}}$ [g]	$m_{m_{h}}$ [g]	$V_{m}$ [cm^3^]	$\rho_{m}$ [g/cm^3^]
MAA 5	0.5529 ± 0.0001	0.1540 ± 0.0001	0.5618 ± 0.0002	0.9841 ± 0.0004
MAA 25	0.5285 ± 0.0001	0.1426 ± 0.0001	0.5435 ± 0.0002	0.9723 ± 0.0004
MAA 37	0.4937 ± 0.0001	0.1429 ± 0.0001	0.4940 ± 0.0002	0.9992 ± 0.0005
SAMAA 5	0.4640 ± 0.0001	0.1285 ± 0.0001	0.4724 ± 0.0002	0.9821 ± 0.0005
SAMAA 25	0.4725 ± 0.0001	0.1276 ± 0.0001	0.4858 ± 0.0002	0.9727 ± 0.0004
SAMAA 37	0.3976 ± 0.0001	0.1149 ± 0.0001	0.3982 ± 0.0002	0.9985 ± 0.0006
ECMAA 5	0.5742 ± 0.0003	0.1687 ± 0.0003	0.5710 ± 0.0002	1.0056 ± 0.0005
ECMAA 25	0.6254 ± 0.0003	0.1713 ± 0.0003	0.6396 ± 0.0002	0.9779 ± 0.0005
ECMAA 37	0.5533 ± 0.0003	0.1575 ± 0.0003	0.5575 ± 0.0002	0.9924 ± 0.0005
MAAG 5	0.4214 ± 0.0001	0.1172 ± 0.0001	0.4285 ± 0.0002	0.9834 ± 0.0005
MAAG 25	0.3919 ± 0.0001	0.1058 ± 0.0001	0.4030 ± 0.0002	0.9725 ± 0.0005
MAAG 37	0.4400 ± 0.0001	0.1273 ± 0.0001	0.4405 ± 0.0002	0.9990 ± 0.0005
SAMAAG 5	0.4261 ± 0.0001	0.1187 ± 0.0001	0.4329 ± 0.0002	0.9842 ± 0.0005
SAMAAG 25	0.4291 ± 0.0001	0.1163 ± 0.0001	0.4405 ± 0.0002	0.9741 ± 0.0005
SAMAAG 37	0.2931 ± 0.0001	0.0834 ± 0.0001	0.2953 ± 0.0002	0.9926 ± 0.0008
ECMAAG 5	0.5675 ± 0.0003	0.1675 ± 0.0003	0.5634 ± 0.0002	1.0073 ± 0.0005
ECMAAG 25	0.5246 ± 0.0003	0.1523 ± 0.0003	0.5244 ± 0.0002	1.0005 ± 0.0006
ECMAAG 37	0.5569 ± 0.0003	0.1588 ± 0.0003	0.5606 ± 0.0002	0.9935 ± 0.0005

**Table 5:** EC growth percentage differences.

Time [h]	% Difference		
	37 °C	25 °C	4 °C
24	18.64	12.84	4.98
52	39.00	106.49	36.82
121	49.01	153.85	38.91

## Data Availability

The original contributions presented in this study are included in the article. Further inquiries can be directed to the corresponding authors.

## Abbreviations

LIIFAMIR^x^	Laboratorio de Investigación e Instrumentación en Física Aplicada a la Medicina
	e Imágenes de Rayos X
MAA	Medium with agar-agar
MAAG	Medium with agar-agar and glucose
SA	*Staphylococcus aureus*
EC	*Escherichia coli*
